# *In-situ* observations and acoustic measurements upon fragmentation of free-floating intermetallics under ultrasonic cavitation in water

**DOI:** 10.1016/j.ultsonch.2021.105820

**Published:** 2021-11-02

**Authors:** Abhinav Priyadarshi, Mohammad Khavari, Shazamin Bin Shahrani, Tungky Subroto, Lukman A. Yusuf, Marcello Conte, Paul Prentice, Koulis Pericleous, Dmitry Eskin, Iakovos Tzanakis

**Affiliations:** a Faculty of Technology, Design and Environment, Oxford Brookes University, Oxford OX33 1HX, UK; b Brunel Centre for Advance Solidification Technology (BCAST), Brunel University London, Uxbridge UB8 3PH, UK; c Cavitation Laboratory, School of Engineering, University of Glasgow, Glasgow G12 8QQ, UK; d Anton Paar TriTec SA, Vernets 6, 2035 Corcelles, Switzerland; e Computational Science and Engineering Group (CSEG), Department of Mathematics, University of Greenwich, London SE10 9LS, UK; f Tomsk State University, Tomsk 634050, Russia; g Department of Materials, University of Oxford, Oxford OX1 3PH, UK

**Keywords:** Grain refinement, Primary intermetallic crystal, Fragmentation time, High-speed imaging, Free-floating

## Abstract

Grain refinement in alloys is a well-known effect of ultrasonic melt processing. Fragmentation of primary crystals by cavitation-induced action in liquid metals is considered as one of the main driving mechanisms for producing finer and equiaxed grain structures. However, in-situ observations of the fragmentation process are generally complex and difficult to follow in opaque liquid metals, especially for the free-floating crystals. In the present study, we develop a transparent test rig to observe in real time the fragmentation potential of free-floating primary Al_3_Zr particles under ultrasonic excitation in water (an established analogue medium to liquid aluminium for cavitation studies). An effective treatment domain was identified and fragmentation time determined using acoustic pressure field mapping. For the first time, real-time high-speed imaging captured the dynamic interaction of shock waves from the collapsing bubbles with floating intermetallic particles that led to their fragmentation. The breakage sequence as well as the cavitation erosion pattern were studied by means of post-treatment microscopic characterisation of the fragments. Fragment size distribution and crack patterns on the fractured surface were then analysed and quantified. Application of ultrasound is shown to rapidly (<10 s) reduce intermetallic size (from 5 mm down to 10 μm), thereby increasing the number of potential nucleation sites for the grain refinement of aluminium alloys during melt treatment.

## Highlights

• Effective ultrasonic treatment domain identified for intermetallic fragmentation.
• Intermetallic breakage occurred in <100 ms from the onset of cavitation.
• High-speed imaging captured shock waves breaking free-floating intermetallics.
• Ultrasonic treatment led to severe cavitation erosion of intermetallic fragments.
• Sono-fragmentation of intermetallics showed significant particle size reduction.

## Graphical abstract

**Figure:**
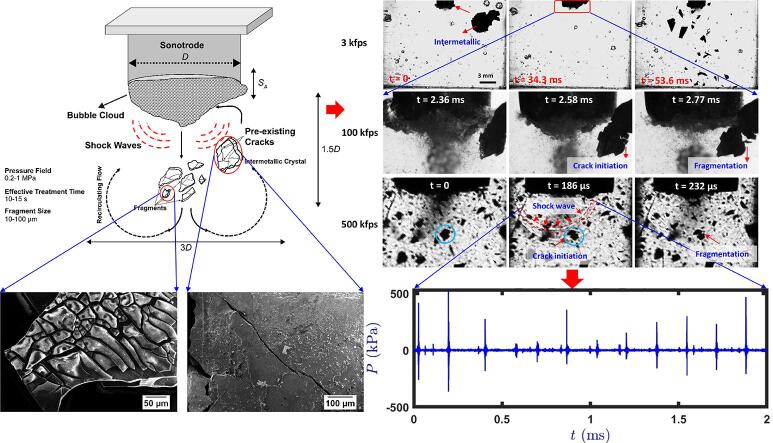

## 1 Introduction

Aluminium alloys are of great importance in many engineering fields such as aerospace, automobile and the military industry owing to benefits comprising high strength to weight ratio, low density and high corrosion resistance. A lot of work has been focussing towards refining the microstructure of as-cast Al alloys to further improve their structural integrity and performance. Ultrasonic melt treatment (UST) has been found very beneficial in this respect, by improving the properties of the as-cast alloys through grain refinement, melt degassing, and improved structure uniformity. UST combines with conventional solidification processes such as direct-chill (DC) casting to offer an environmentally friendly and cost effective treatment [1], [2], [3]. Although UST is a well-established technique in processing melts [1], [4], the exact mechanisms of grain refinement are still under scrutiny. There are two commonly recognised mechanisms of grain refinement acting upon conventional casting: enhanced heterogeneous nucleation on activated non-metallic inclusions through improved wetting and the sono-capillary effect and, fragmentation of primary intermetallics/dendrites through cavitation-induced shock waves [1], [5], [6]; with the latter process being very efficient and generally known as sono-fragmentation. Application of ultrasound to achieve particulate refinement has also been widely studied in the areas related to sonocrystallisation and precipitation processes [7], [8], [9], [10]. The phenomena of potential refinement of such particulates have previously been ascribed to stable-inertial bubble implosions, high pressure shock waves (order of GPa), powerful microjets and enhanced mixing of particles [7], [11], [12]. However, no concrete evidence of the same were provided leaving it as a hypothesis yet to be validated. Recently, a number of studies have been made to discern the ultrasound-induced fragmentation dynamics of various dendrites and crystals by application of in-situ synchrotron X-radiography imaging [13], [14], [15], [16]. All these studies were done on fixed dendrites or intermetallic crystals. Moreover, real-time capturing of cavitation bubble dynamics and their interaction with solid phases in an actual melt system has been constrained due to working temperatures, opacity and temporal and spatial limitations of even the most advanced X-ray imaging [16]. This calls for alternative analysing approaches, whereby detailed cavitation dynamics can be recorded and resolved in-situ. A suitable transparent candidate for such studies is water. Water shares similar cavitation behaviour with liquid aluminium, though the acoustic pressure can be up to 2 times larger in Al melts [17], [18]. For this reason it has been systematically used to replicate the conditions of acoustic cavitation in liquid Al during UST [19], [20], [21].

To this end, numerous studies have been performed using water or other transparent organic solutions to capture the fragmentation mechanism of solid phases. For example, the effects of UST on solidification were visually investigated in water [22] and in different organic compounds such as camphene [23], succinonitrile-camphor [24] and sucrose based solutions [25], [26] where it was observed that an oscillating bubble cloud and shock waves arising from violent bubble collapses were primarily responsible in fracturing growing dendrites and intermetallics. Additionally, in-situ synchrotron observations revealed that the induced acoustic flow can also play a major role in the redistribution and fragmentation of growing dendrites [16]. Intermetallics play a significant role in the formation of Al alloys structure and properties. Their fineness and uniformity of distribution are of great importance, as large particles are detrimental for mechanical properties acting as stress concentrators. The majority of intermetallic compounds are deemed to be structurally strong at ambient temperatures and maintain their hardness with increasing temperature [27], [28] without drastic change up to the melting point (660 °C) of aluminium, though the specific properties such as strength and ductility of Al-based intermetallics at high temperatures cannot be found in literature. Nevertheless, the use of transparent media at ambient temperature seems appropriate for elucidation of the fragmentation mechanisms involved. Very few studies related to fragmentation of primary intermetallics has been reported until now. Wang et al. [29] recently studied in-situ fragmentation of various fixed primary crystals such as Al_3_Ti, Si and Al_3_V subjected to ultrasound in water. Also the fragmentation of fixed-in-space Al_2_Cu primary intermetallics in aluminium melt subjected to UST was observed upon in-situ synchrotron studies [13]. Further works [22], [30], [31] have concluded that shock waves generated by cavitation bubble collapses are predominantly responsible for the fragmentation of primary intermetallics. Fragmentation of these crystals to appropriate shape and sizes is also vital for an effective grain refinement [32], [33]. In addition, these refined crystals may act as particulate reinforcement in metal matrix composites (MMC’s) significantly improving their physical, mechanical and, chemical properties such as elastic modulus, hardness and thermal stability [1], [34]. However, due to limitations of studying fixed crystals or having a small field of view in X-ray radiography, understanding the combined role of acoustic cavitation and streaming in the overall refinement process remains elusive and, therefore, calls for the need of further research in this regard.

The research presented here builds on our previous study [22]. Here we aim to first characterize the fragmentation process conditions in terms of breakage time and associated pressure field, using fixed intermetallic crystals and then move on to more realistic conditions of intermetallic fragmentation that represent the real case scenario in liquid metal during UST, i.e. identifying the fragmentation mechanism in the case of free-floating crystals within a dynamic cavitating environment. The latter has not been done before, to the best of our knowledge. Quantifying the cavitation-induced fragmentation of floating dendrites and primary intermetallics and finding their temporal and spatial fragmentation patterns are crucial in realising the optimal treatment conditions for effective up-scaling of the UST process. It is known that these crystal fragments act as source of heterogeneous grain nucleation points in real melt system subsequently leading to grain refinement and optimum equiaxed structure [13], [33], [35], [36], [37]. The role of Al_3_Zr in the grain refinement of aluminium alloys upon UST has been established elsewhere [38]. Wang et al. [37] observed the formation of primary Al_3_Zr particles in Al-0.4% Zr alloy after UST was applied at different temperature ranges. Their study mainly focussed on the particle refinement under the influence of UST. It has been reported that grain refinement in ultrasonically treated Al alloys is dependent on the nucleation potential and number density of primary intermetallic particles [39], [40], [41]. Another important characteristic of UST-induced grain refinement is that the fragmented dendritic crystals can also significantly improve the microstructure, homogeneity, and quality of the casting, without the need of substantial amount of grain refining particles or addition of AlTiB master alloys [42]. Jung et al. [43] reported that primary phase particles of the order of 10 μm or less was sufficient for α-Al nucleation. The final microstructure observed after solidification of treated melts is therefore systematically connected with the refinement capability of the formed primary phases. Although, these investigations provided a general overview of the microstructural refinement obtained after the UST process, there is no evidence yet available in the literature that quantifies the time-based size-reduction potential of the intermetallic crystals. These studies also seldom provided the statistical data that would eventually lead to a clear criterion of fragmentation and did not report the extent of effective treatment domain that is essential for the fragmentation response to UST. Understanding of the primary crystal fragmentation potential under the influence of ultrasound by outlining the temporal and spatial behaviour of these fragments thus becomes vital in predicting actual grain refinement in Al based alloys.

The present work, therefore, focuses on firstly determining the extent of the treatment domain in terms of fragmentation time for a fixed crystal subjected to acoustic pressures measured across the treated liquid volume. Subsequently, the effect of ultrasonic treatment on the mechanism of floating-crystal fragmentation under various sonication periods in de-ionised water (DIW) is investigated using in-situ real-time high-speed imaging and post-mortem characterisation.

## 2 Materials and equipment

### 2.1 Sample preparation

An Al-3 wt% Zr alloy was produced by smelting commercially pure Al (99.97%) and a master alloy (Al-5 wt% Zr) and cast into a 350 g ingot. The ingot was then re-melted in an electric furnace and subsequently slowly cooled in a graphite crucible (50 mm diameter, 150 mm height) inside the furnace as per the cooling cycle described elsewhere [22], [30], [31]. The solidified ingot was then cut into multiple cubes (5 × 5 × 5 mm) using a rotating SiC blade. These cubes were then submerged in a 15% water solution of caustic soda (NaOH) for about 24 hrs to dissolve the Al matrix and extract Al_3_Zr crystals from the alloy.

**Fig. 1:**
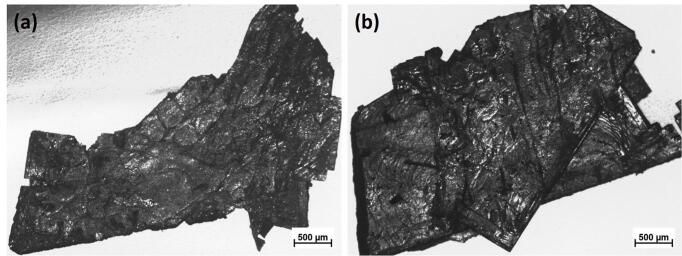
Optical micrographs of typical extracted primary Al_3_Zr crystals.

The crystals were then collected after filtering out the solution and were carefully rinsed using ethanol. They were subsequently left to dry out for microscopic observations. Fig. 1 shows optical images of a typical extracted crystal before exposure to ultrasound. The dimensions of this particular crystal were L: 3 ± 1 mm × H: 5 ± 1 mm × W: 0.06 ± 0.01 mm. Numerous similar crystals were collected and used in the subsequent experiment. The evolution of the crystals’ morphology during ultrasonic treatment will be discussed in detail in Results and Discussion.

### 2.2 Experimental setup

The following experiments were strategically designed to quantify the fragmentation behaviour for fixed and a free-floating primary intermetallics. Firstly, the treatment domain defined as the region where fragmentation occurs within a very short period of time (<100 ms) was outlined for a fixed intermetallic at different input powers of ultrasound. The treatment domain was also effectively characterised through maximum acoustic pressure mapping across the cavitation field by using a calibrated needle hydrophone system. Thus by observing the fragmentation process while monitoring the acoustic pressure field, we could resolve the effective range of pressure amplitudes where fragmentation occurred. Secondly, the crystal break-up and the flow pattern resolved by the floating fragments were captured in-situ by high-speed imaging. The cavitation erosion pattern of free-floating primary crystals was characterised with respect to the sonication period using scanning electron microscopy (SEM). Finally, the number and size distribution of fragments were subsequently evaluated at different sonication time intervals.

#### 2.2.1 Fixed intermetallic

An extracted primary crystal was first fixed on top of a steel substrate as described in [22] and positioned at the base of a glass container with dimensions L: 75 mm × W: 75 mm × D: 100 mm. The container was filled with DIW to a height of 75–80 mm and was then placed below a 500 W piezoelectric horn transducer (Hielscher UIP500hdT) operating at 20 kHz. The ultrasonic waves were introduced into the water-filled container through a cylindrical titanium sonotrode (Ø = 22 mm) immersed approximately 10 mm below the water surface. The driving amplitude of the vibrating sonotrode was chosen to run at 3 different power levels: 11.4 μm peak to peak (p-p) corresponding to 24 W (20%), 34.2 μm p-p to 65 W (60%) and 57 μm p-p to 108 W (100%). The experiments were conducted in ambient (∼25 °C) conditions. After each experimental run, a fresh crystal was placed across varying locations in transverse (X-axis) and longitudinal (Y-axis) directions with co-ordinates, 0 ≤ x ≤ 30 mm and 5 ≤ y ≤ 30 mm, respectively at a gap of 5 mm between two consecutive positions. A schematic of the experimental setup is shown in Supplementary Fig. S1. The fragmentation time of the crystal leading to first breakage from the onset of cavitation was obtained. At least 2–3 readings were taken for each position of the crystal for reproducibility of the results. The actual fragmentation time and boundaries of the effective treatment domain where crystals seemed to be prone to breakage by the action of ultrasound were defined.

Acoustic emissions were recorded with a 4-mm PVdF Needle Hydrophone (NH) and a 125-μm Fibre Optic Hydrophone (FOH) sensor (Precision Acoustics Ltd.) calibrated in the range of low (8–400 kHz) and high (1–30 MHz) frequencies, respectively, with the sensitivity function shown in Supplementary Fig. S2 (a, b). The acoustic measurements were captured across the treatment domain by using NH at each of the defined co-ordinates (5 ≤ x ≤ 30 mm, 5 ≤ y ≤ 30 mm) as previously described for the fixed position (Fig. S1). In addition, synchronised acoustic emissions and in-situ fragmentation imaging measurements were recorded by placing the FOH sensor in the vicinity of a fixed crystal to monitor as accurately as possible the shock wave pressure amplitude required for fragmentation. The raw voltage data were acquired with a Peripheral Component Interconnect (PCI) data acquisition device with sampling rate of 20 × 10^6^ samples/s which allowed real-time signal tracking and recording of high-speed images and the FOH data. Fast Fourier transformation was applied to the voltage–time data to obtain the pressure values following the deconvolution process defined in our earlier work [17], [18], [44] and in [45]. For repeatability of the results, the experiments were conducted at least 4–5 times for each position and input power, including 3 extra runs without the presence of intermetallics.

#### 2.2.2 Free-floating intermetallic

An extracted primary Al_3_Zr crystal was placed in a transparent cuvette of dimension L: 25 mm × W: 10 mm × D: 45 mm containing 4 ml of DIW and allowed to settle at the bottom. The container was then placed below a 200-W piezoelectric ultrasonic transducer (Hielscher UP200S) operating at a frequency of 24 kHz. Detailed specification of this ultrasound device can be found elsewhere [46]. In this case, a titanium tapered sonotrode with a tip 3 mm in diameter was used that allowed for detailed observations of the highly dynamic bubble/ fragment interactions. The sonotrode-tip was vertically submerged into the liquid up to a depth of 5 mm to sonicate the medium at room temperature conditions (25 °C). The liquid was sonicated for different time intervals: 3, 6 and 9 s from the onset of cavitation with a sonotrode-tip vibration amplitude (*S_A_*) fixed at 210 μm peak to peak.

The in-situ fragmentation of the free-floating intermetallic crystals upon ultrasonication was captured using Photron SA-Z Fastcam high-speed camera (Photron Inc.). The camera was combined with Navitar 12 × adapter lens and nominal frame rates of 3,000 and 100,000 frames per second (fps) were chosen to capture the whole fragmentation sequence with imaging resolution of 640 × 512 pixels and 384 × 256 pixels, respectively. The background illumination was supplied by a multi LED flash lamp (GS Vitec) arranged from the rear of the container. A schematic illustration of the in-situ high-speed imaging setup is shown in Supplementary Fig. S3 (a). The crystal fragmentation was repeated 5 times, with a new extracted crystal used each time. In this paper, we only show the most illustrative sequence of images with corresponding videos (in Supplementary Material). After sonication, the liquid was filtered over a 2.5-μm pore-sized filter paper to separate the fragmented crystals. The particles were then air dried and preserved for SEM examination. The crystal velocity and size distribution during and after sonication, respectively was measured using image tracking processing techniques by ImageJ 1.8.0.

To capture the sono-fragmentation of a crystal by the propagating shock waves emanating from cavitation cloud collapses beneath the horn-tip, shadowgraphic imaging (Supplementary Fig. S2 (b)) was performed using an ultra-high-speed camera (HyperVision Shimadzu, Japan) with a resolution of 450 × 200 pixels. In order to resolve the emitted shock waves, a similar Schlieren imaging setup was used as discussed elsewhere [22], [44], [47]. The fragmentation sequence was captured with high temporal resolution of 500 kfps over a short period of 512 μs. The spatial resolution of 17 μm/pixel was obtained using a Milvus 100-mm macro lens (Zeiss, Oberkochen, Germany) with 10 ns synchronous laser pulse illumination (CAVILUX Smart, Cavitar, Finland) coupled to a collimating lens.

## 3 Results and Discussion

### 3.1 Fragmentation of a fixed crystal and characterization of the treatment domain

In a real liquid metal system subjected to UST, it is essential that the entire melt volume undergoes treatment. Therefore, identifying the borders of the effective treatment domain becomes very important to ensure the effectiveness of ultrasonic treatment on crystal fragmentation, in order to optimise the processing of large melt volumes by using a single energy source (single sonotrode) [42], [48]. In this section, we outline the spatial treatment domain in terms of the time required to fracture a single fixed crystal and relating it to the acoustic pressure field generated by the ultrasound.

**Fig. 2:**
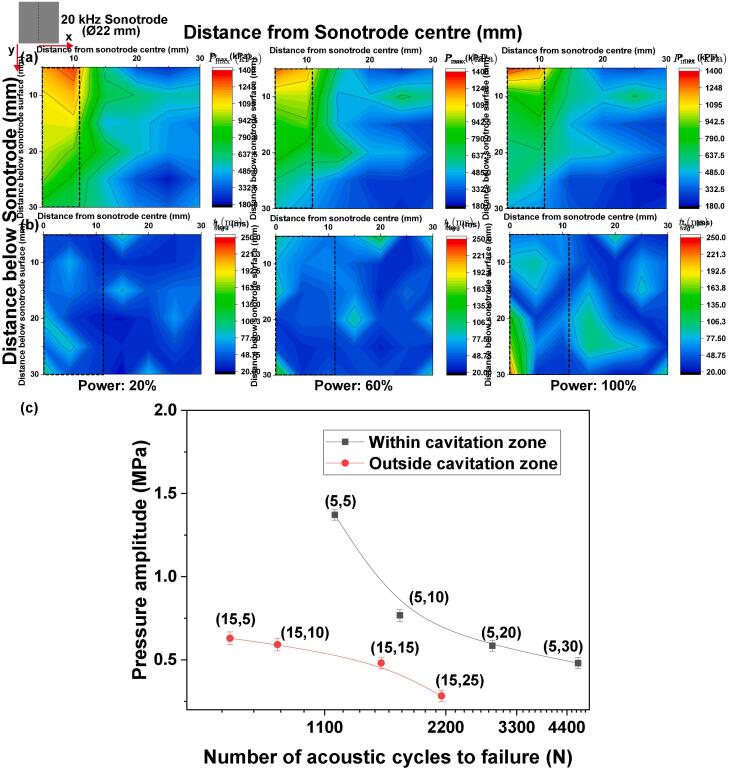
Contour plots of (a) effective treatment domain with maximum pressure (P_max_) distribution obtained from NH, (b) fragmentation time of fixed intermetallic crystals, obtained at 20%, 60% and 100% input power. (c) Variation of cyclic pressure amplitude with respect to the acoustic (sonotrode motion) cycles to failure of the crystal measured from the onset of cavitation activity at 100% input power at (x, y) position in mm away from the horn.

Figure 2a and b show respectively the 2D mapping of the measured maximum acoustic pressure (*P_max_*) obtained using a NH at specific positions as described in section 2.2.1 and the associated crystal fragmentation time (*t_frag_*) distribution for different sonotrode amplitudes as mentioned in section 2.2.1. The sizes of all the crystals that were subjected to cavitation action from ultrasound were in the range of 3–5 mm. For clarity, we define the cavitation zone as the area within the limits of the tip of the sonotrode (marked as the rectangular region below the horn-tip in Fig. 2(a, b)) where the cavitation cloud is formed i.e., up to a distance of 11 mm away from the centre of sonotrode with pressure amplitude fluctuating between 600 and 1300 kPa along its borders. The region beyond that is referred as outside the cavitation zone. The fragmentation time of the crystal was measured by placing the intermetallics within and outside the defined cavitation zone in order to check the effective treatment volume depending on the position and operating transducer power. As it can be seen in Fig. 2b, the fragmentation time of a crystal remains largely unaffected throughout the treatment domain measured within the cavitation zone (x < 11; 5 ≤ y ≤ 30 mm) irrespective of transducer powers of 20% and 60% and varies in the range of 20–100 ms from the cavitation inception. However, for 100% power, the fragmentation time dramatically rises from 80 ± 10 ms at 20% up to 240 ± 30 ms in the location ×  = 0 mm and y = 30 mm below the sonotrode surface. The reason for this discrepancy could be the developed acoustic shielding effect [49], [50], [51] caused by the dense bubble cloud formation near the sonotrode or due to the extended periods of non-collapsing bubble deflations [47]. For crystals positioned immediately outside the cavitation zone (11 ≤ x ≤ 30; 5 ≤ y ≤ 30 mm), there is only about 5–10% increase in the fragmentation time of the intermetallic crystals. The measured acoustic pressure (Fig. 2a) on the other hand is highest in close proximity to the sonotrode surface and attenuates with increasing distance away from the source as expected. The maximum pressure outside the cavitation zone varies between 180 and 1000 kPa for transducer powers operating at 20%, 60% and 100%. Even with the attenuation of the acoustic pressure, the fragmentation time remains mostly unaffected immediately outside the cavitation zone. For regions farther from the active treatment domain (x > 30; y > 30 mm), no fragmentation was observed, possibly owing to reduced acoustic pressures. It is interesting to note here that the fragmentation of these crystals is effectively occurring both inside and outside the cavitation zone which means that the breakage response is also somewhat dependent on the physical condition of the material as explained below.

It is usually expected that fragmentation will occur faster at higher pressure amplitudes and closer to the ultrasonic source. However, this was not the case in our experiments. Almost all intermetallic crystals broke within 100 ms of cavitation onset and the fragmentation time did not vary much with the position of a crystal for the 20% and 60% transducer power. This interesting behaviour may be attributed to the morphology of intermetallic crystals. Al_3_Zr intermetallics are highly brittle in nature with the fracture toughness close to 1.1 MPa√m [22] and usually contain many micro-cracks/defects formed during solidification that act as stress concentration points resulting in failure of the material upon continuous interaction with shock waves emitted from the cavitation bubble collapses. It has been previously reported that crystal failure under the presence of acoustic cavitation occurred through a fatigue mechanism. During certain number of acoustic pressure cycles, the micro-cracks present within the crystal propagate until the critical crack size is reached leading to its brittle fracture [22]. In the case of 100% power, however, the fragmentation time increased by a factor of 2–3 compared to 20% and 60% input power, with the breakage time being about 250 ms farthest below the horn (y = 30 mm). This is better depicted in Fig. 2c where the plot of pressure amplitude versus the number of acoustic cycles obtained for the 100% input power indicates that the number of acoustic cycles to failure inside the cavitation zone is almost 2 times higher than outside the cavitation zone for each depicted position (x, y). This can then be attributed to strong shielding (due to formation of a larger cavitation cloud beneath the sonotrode) or extended non-collapsing periods of the numerous bubbly structures as previously discussed that obstruct and cushion the propagation of shock wave emissions, which are known to be the driving force for fragmentation of intermetallic crystals [30], within the cavitation zone. This also causes the suppression of the pressure field within the cavitation zone at 100% compared to 20% as seen Fig. 2a. Thus, even though the pressure amplitude (predominantly associated with the fundamental/driving (f_0_), subharmonics, harmonics, and ultra-harmonics arising from the cavitation activity) is of the same order as in case of 20% and 60% input power, the fragmentation time is actually larger. This is however not the case outside the cavitation zone, where there are always some prevailing horizontal components of the propagating shock waves generated from the edges of the sonotrode that remains unaltered as previously observed in [22], [44]. Therefore, based on the fragmentation time and acoustic pressure measurements obtained from NH (Fig. 2(a, b)), and since the measured pressure field, in the range of up to 400 kHz, spans within the same order of pressure amplitude for all input powers, it can be hypothesised that shielding of shock waves at the higher input power (100%) may be the reason for increase in the critical fragmentation time within the cavitation zone.

For that reason, the pressure field generated from the shock wave emissions was further studied using the FOH probe [44] calibrated in the MHz range (see section 2.2.1) through synchronised high-speed imaging of crystal fragmentation and shock pressure measurement setup as described in section 2.2.1. This allowed us to test this hypothesis and verify if the cloud formation with the parallel suppression of shock waves is the main reason for the delay of the fragmentation.

**Fig. 3:**
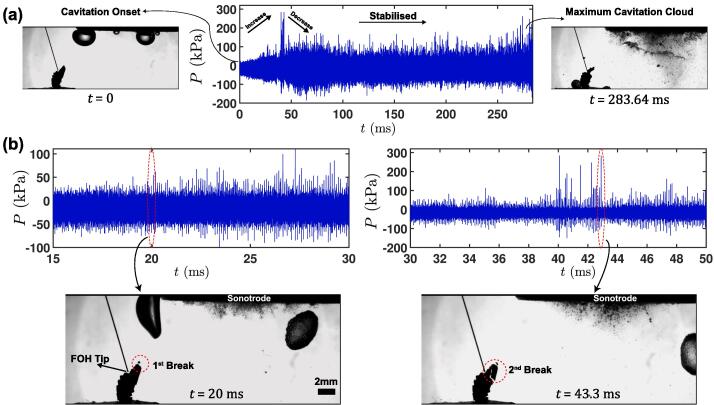
Synchronised capture of in-situ high-speed imaging and acoustic pressure obtained by the high frequency calibrated FOH sensor; (a) pressure–time profile indicating the onset of cavitation and maximum bubble cloud, (b) the crystal breakage snapshot with the corresponding pressure peak. Supplementary Video 1 can be accessed with the online version of the article.

Figure 3 (Supplementary Video 1) shows the fragmentation process of a fixed crystal with the dimensions L: 2.4 mm × H: 4.2 mm × W: 0.06 mm positioned 10 mm below and 15 mm in the transverse direction from the centre of the sonotrode operating at 42 μm p-p tip amplitude. Fig. 3a shows the pressure–time profile captured from the onset of cavitation (t = 0 ms) up to maximum bubble cloud formation (t = 283.64 ms) obtained from the FOH sensor tip placed near the crystal. Once the cavitation initiates, the shock pressure peaks started to appear with gradual increase in the pressure amplitude. This continuous exposure to shock waves, indicated by the sharp pressure peaks from the periodic implosions of cavitation cluster below the horn-tip arguably generated low-cycle fatigue within the material, leading to crystal fragmentation [22]. Fig. 3b shows the shock pressure peak and the corresponding fragmentation snapshot of crystal at t = 20 ms and t = 43.3 ms when a bundle of high intensity shock waves is emitted. The major fragmentation occurs at t = 43.3 ms when the shock wave intensity was the strongest with the peak positive pressure amplitude about 300 kPa. Along with this major breaking event, various smaller fragments were also formed simultaneously from the fractured region indicative of the typical brittle material failure (see Supplementary Video 1). Interestingly, just before the 2nd breakage occurred, a small bubble was seen to grow and oscillate close to the crystal fracture location and the FOH tip. However, in our opinion the collapsing bubble had no role to play in the fragmentation of the crystal in this case, as these tiny cavitation bubbles cannot produce pressure surges of the magnitude that is responsible for the breakage of an Al_3_Zr intermetallic. Even if it did, the acoustic cycles required to break the crystal should be significantly higher as previously discussed in Priyadarshi et al. [22], [29]. Also, the rise of shock pressure at the same instant as the bubble collapses and the fragmentation occurs can be purely coincidental. Supplementary Video 2 shows a high-speed video recording demonstrating the fragmentation response of a primary crystal captured under same experimental conditions as done for Supplementary Video 1. From Video 2, it can be clearly seen that the 1st fragmentation event occurred just after the inception of cavitation at the horn tip followed by the 2nd breakage and also there was no bubble oscillating close to the crystal or the FOH tip this time. This further shows that the shock waves are the primary reason for the intermetallic fragmentation as also reported earlier [22]. Moreover, as observed from Fig. 3 (Supplementary Video 1), the crystal breakage happened within 100 ms of the onset of cavitation and much before the fully developed cavitation regime was reached. It should be noted that after the 2nd breaking event, no further fragmentation was observed until much later, i.e. ∼ 284 ms following the onset of cavitation. It is apparent that the developed cavitation cloud beneath the sonotrode-tip shields the propagation of shock waves causing the pressure amplitude to supress as seen in Fig. 2a (100%) and previously observed in other studies [44], [52]. Thus, the subsequent fragmentation takes much more time to occur until the existing crack/flaw size reaches its critical length.

As shown in Fig. 2b, the treatment domain that was responsible for crystal fragmentation extended up to 30 mm from the sonotrode axis in both the vertical and horizontal plane. With taking into account the 22 mm diameter of the sonotrode, this falls within the well-known 1.5D rule of thumb for the cavitation zone [53]. It should also be noted that the fragmentation occurred well before the cavitation cloud under the sonotrode ever reached its maximum size (Fig. 3, Video 1). Therefore, the crystal breakage occurring between 20 and 100 ms after cavitation inception can be regarded as the effective treatment period caused by high intensity shock waves emissions, as long as the crystals were within the fragmentation domain (x < 30; y < 30 mm) at low input powers, where shock waves obstruction is supressed. Upon real cavitation in liquid metals, there should be a trade-off between the input power and bubbles formation (cavitation activity) that define the effective treatment domain as has been previously highlighted in [54].

### 3.2 Observation of free-floating crystal breakdown

After identifying the actual fragmentation zone and relating it to the corresponding acoustic pressure field and dynamics of shock waves, the next step is to monitor and reveal the fragmentation process of free-floating intermetallics. These primary intermetallic crystals that are formed in real metallic melts are typically free-floating under conventional UST. Thus, the fragmentation dynamics of these primary crystals is governed not only via the ultrasonic cavitation regime but also through the developed acoustic flow streaming within the liquid medium. The following section discusses the breakage mechanism of a free-floating intermetallic particle in the liquid irradiated with power ultrasound.

**Fig. 4:**
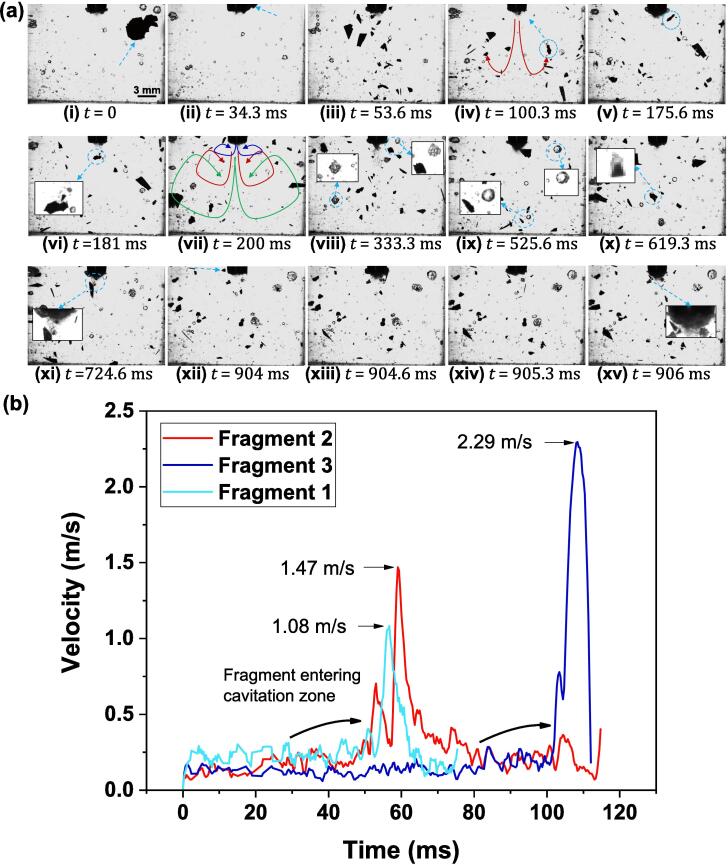
(a) High-speed image sequence showing the break-up mechanism of free-floating primary crystal recorded at 3000 fps. The corresponding videos 3, 4 and 5 can be accessed with the online version of the article. (b) Measured instantaneous velocity–time profile of recirculating intermetallic fragments flowing towards and through the cavitation zone. Supplementary Video 7 can be accessed with the online version of the article.

Figure 4a (i) shows a faceted crystal (marked with an arrow) with approximate dimensions of L: 5.2 mm × H: 4.1 mm × W: 0.06 mm freely floating in water at time, t = 0 ms. Forced convection induced by acoustic flows prevented settling of this particle. After about 34 ms, the crystal driven by the streaming forces reached neat the horn-tip as shown in Fig. 4a (ii). As soon as the crystal approaches the cavitation cluster formed beneath the vibrating horn, catastrophic fracture “instantly” occurred with it disintegrating into small fragments as shown in Fig. 4a (iii). Subsequently, the fragmented crystals were seen to follow a recirculating streaming path, for example the encircled crystal in Fig. 4a (iv) flowed back towards the cavitation zone (Fig. 4a (v)) thereby coming into contact with the bubble cloud again causing the crystal to further break down into smaller fragments, as shown in Fig. 4a (vi) (marked with an arrow). Some of the fragments tended to accelerate after interacting with the strong cavitation cloud formed beneath the sonotrode (see Supplementary Video 3 for clarity). The velocity of fragments after passing through the cavitation zone varied in the range 1–2 m/s (see more on that below). Note, the recirculation flow pattern may be different for each fragment depending on its size and initial momentum, and may also change course to follow different paths as shown in Fig. 4a (vii). Nevertheless, it is certain that each and every fragment will follow some acoustic flow pattern and will pass through the strong cavitation region multiple times during its motion. It is also evident from Video 3 that some of the fragments did not come in contact with the cloud and remained unaffected. In addition, multiple small and large cavitation bubbles were also observed vigorously coalescing, splitting and changing their oscillating pattern surface (Faraday waves) continuously as shown in Fig. 4a (viii-ix) (see Supplementary Video 4). These bubbles were also seen moving against the streaming motion, attaching themselves to the fragmented particles (Fig. 4a (x-xi)) and transporting them back to the cavitation zone (see Supplementary Video 5). Further breakage of crystal fragments that recirculate back to the cavitation zone was almost instantaneous and occurred within 2 ms (Fig. 4a (xii-xv)). It is evident from Supplementary Videos 3–5 that only some of the crystal fragments undergo further breakage after recirculating back towards the vibrating source. We also did not observe any fragmentation of smaller-sized crystals as capturing such fine details could be constrained by the optical resolution and depth of field limitation of the high-speed camera. The role of acoustic streaming as seen here was mostly to promote the recirculation of crystals back to the cavitation zone (vicinity of the cavitation cloud beneath the horn tip) for further treatment where the acoustic pressure intensity is highest. It should be however noted that the floating fragments when entering the cavitation zone did not break by coming into direct contact with the vibrating horn as the tip was covered by the cavitation bubble clouds for almost all the sonication period, even during the compression cycle of the vibrating source. Other possibilities of the fragments breaking through crystal – crystal, crystal – horn and crystal – wall interaction were also ruled out elsewhere [12], [55]. It can be seen in Supplementary Video 6 recorded at 5 kfps that a large crystal approaching the ultrasonic source rebounded and did not break even after colliding with the horn surface, validating the observation made by Zeiger et al. [12]. It has been found that the fragmentation response of materials during ultrasonic treatment of different solid–liquid phase systems essentially depends on the type of material being sonicated. In the case of softer metals, the particle – particle collision leads to surface deformation and interparticle melting [56], [57], [58]. Whereas, in the case of hard brittle materials like in our study, the fragmentation of materials is primarily through direct particle – shock wave interaction [12]. However, to be able to attribute such mechanism to the case of sono-fragmentation of primary intermetallics, we have performed high-speed imaging at high frame rates further below in this section in order to validate this hypothesis.

Fig. 4b shows the velocity–time profile obtained after real-time tracking of three free-floating fragments (see Supplementary Video 7) with sizes of the order of 1.48 ± 0.8 mm with Fragment 1 being the largest and 3 being the smallest; as they come into the vicinity of the cloud beneath the oscillating horn and pass through the cavitation zone. It can be seen from Fig. 4b that the average instantaneous velocity of the fragments before interacting with the cavitation zone was approximately 0.34 ± 0.1 m/s, consistent with acoustic flows based on previous Particle Image Velocimetry (PIV) measurements [59]. As soon as the fragments entered the cavitation zone, their velocity rapidly increased to 1.6 ± 0.5 m/s due to the instant push from violent cloud collapses. This change of the velocity reflected the collapse of large cavitating cloud that imparted a strong shock impulse (rate of change in momentum) to the nearby fragment inducing sufficient stress to cause particle breakage.

As observed in Fig. 4a (vii), vortices formed on the sides of the main cavitation flow stream brought the fragments back towards the sonotrode as demonstrated by their motion monitored up to 15 mm below the tip of the sonotrode. Acoustic streaming in liquid metals exhibits a typical acoustic flow pattern with a strong downstream jet motion along with a toroidal vortex surrounding the jet. According to [19], [60], for sonotrode operating at 20 kHz in transparent liquids such as water with p-p amplitude of 51 μm, the average acoustic streaming velocity near the tip can reach up to 0.5 m/s. The presence of dominant vortices developed near the cavitation zone was shown to maximise the entrainment and enhanced treatment of floating phases [19]. A similar pattern in the acoustic streaming profiles was also observed in other transparent liquids with such different physical parameters as water and glycerine [20]. UST of an Sn-30 wt% Cu alloy performed during the solidification revealed that the generated acoustic streaming helps to draw the fragmented dendrites back into the melt bulk, thus confirming that disintegration and distribution of solid phases by acoustic cavitation and streaming led to grain refinement in metallic alloy melts [19]. Wang et al. [13] also confirmed that acoustic streaming played a significant role in the fragmentation of a primary solid phase. It was found that the dendritic crystals tended to vibrate upon ultrasonic irradiation and were subsequently detached by the acoustic flow. These crystals were then propelled back into the cavitation zone following the upward path of the recirculating loop where they underwent further breakdown caused by violently imploding bubbles thereby, producing smaller fragments that would act as nucleation sites for secondary phase solidification leading to microstructural refinement. Lebon et al. [59] earlier reported that the acoustic streaming response in water and liquid aluminium is very similar. Although the specific flow and acoustic patterns in the setup used and in metal casting moulds are different, we do not expect large differences in the underlying mechanisms that happen on microscopic rather than macroscopic scale. Note that depending on the acoustic streaming path (as shown in Fig. 4a (vii)) followed by the crystal, the time taken by each fragment to re-enter the cavitation zone can vary from 17 to 120 ms (Fig. 4b). However, the majority of the fragments took close to 100 ms to recirculate back to the cavitation zone following their respective trajectories as shown in Fig. 4a (vii). This is in very good agreement with recent investigation by Beckwith et al. [3], where it was shown that the effective residence time in a real case scenario of UST performed in direct-chill casting launder was close to 100 ms meaning that every fragment should essentially recirculate almost 50 times before moving downstream towards a hot top mould. Therefore, the synergy of strong collapses inside the cavitation zone and adjacent to the sonotrode-tip, in conjunction with the rapid collision of fragments with the bubble cloud may lead to crystal breakage after few acoustic cycles.

**Fig. 5:**
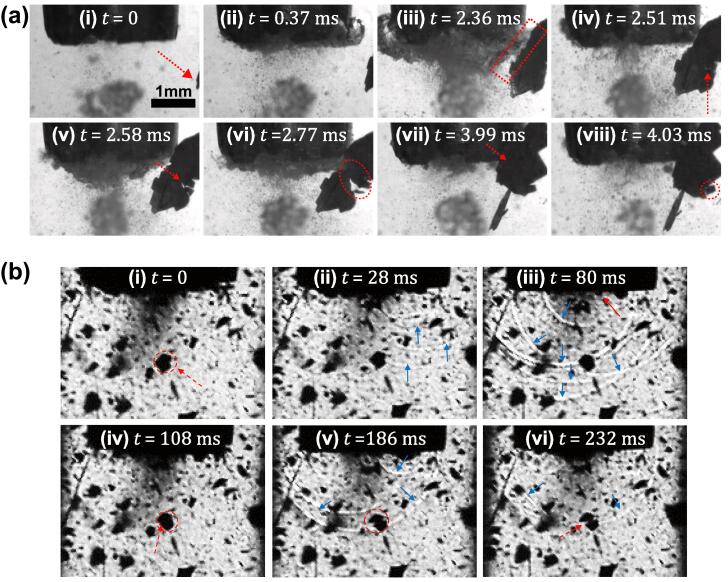
(a) High-speed image sequence showing the crystal break-up mechanism near the cavitation cloud recorded at 100 kfps. Supplementary Video 8 can be accessed with the online version of the article. (b) Series of in-situ high-speed shadowgraphic imaging showing the crystal fragmentation caused by multiple shock wave impacts in the vicinity of cavitation cloud recorded at 500 kfps. Supplementary Video 9 can be accessed with the online version of the article.

In order to clarify the exact fragmentation mechanism of free-floating crystals recirculating and re-entering the cavitation zone, high-speed imaging at higher frame rates, under visible light and laser illumination was carried out. Fig. 5a (i-viii) shows a sequence of images captured at 100 kfps with a zoomed view on the fragmentation of a floating crystal as it approaches the sonotrode. At t = 0 ms, the crystal was seen to flow towards the vibrating sonotrode as shown in Fig. 5a (i). The crystal then started to vibrate with its frequency matching the oscillating frequency (24 kHz) of the cavitation cloud collapse at t = 0.37 ms (Fig. 5a (ii)). As the crystal approached the tip of the sonotrode, the amplitude of crystal vibration increased with its oscillating frequency reducing to 5 kHz equivalent to the *f_o_/4* subharmonic collapse of primary bubble clusters [47]. At t = 2.36 ms, the crystal experienced the maximum deflection and almost came in contact with the cavitation cloud when it was at its maximum size (Fig. 5a (iii)). With the bubble cluster collapsing at t = 2.51 ms, the crack initiated in the crystal and propagated until a section broke off at t = 2.77 ms as shown in Fig. 5a (iv-vi). The remaining parent crystal however continued to flow towards the ultrasonic source and again almost touched the cloud at t = 3.99 ms (Fig. 5a (vii)). Soon after, the crystal was deflected away as the cloud started to grow and another small breakage occurred at t = 4.03 ms (Fig. 5a (viii)). This whole sequence of images can be clearly seen in Supplementary Video 8. It was suggested that the shock waves emitted from these subharmonic cluster collapses are responsible for the fragmentation. To verify this, another set of high-speed imaging experiments (at a higher rate of 500 kfps), represented schematically in Supplementary Fig. S3 (b), was performed to confirm that crystal breakage is caused by the shock waves close to the ultrasonic source.

Figure 5b (i-vi) outlines the series of images showing the crystal breakup occurring near the horn-tip caused by the propagating shock waves (see Supplementary Video 9). Initially (t = 0 μs), a crystal (circled in red) was seen floating near the source (Fig. 5b (i)). As the primary bubble cluster beneath the sonotrode started to collapse, multiple fronted shock waves were released (marked by blue arrows) and incident to the fragmented crystal (Fig. 5b (ii)). Upon complete collapse of the bubble cluster as shown with a red arrow in Fig. 5b (iii), another set of shock waves was released (blue arrows). Frame-by-frame analysis indicates that around 10 shock waves were emitted for every 50 μs (∼1 acoustic cycle). After exposure to around 20 shock waves, delamination seems to occur followed by crack inception on the side of the floating crystal (marked with an arrow in Fig. 5b (iv)). The crystal then eventually broke as the crack propagated further ultimately causing its complete fragmentation at t = 232 μs (Fig. 5b (v-vi)). It should be noted that the direction of the shock wave propagation with respect to the crystal position or point of defect (pre-existing micro-crack) may contribute to the fragmentation process.

The observations of Fig. 4a, Fig. 5a and b reveal that sono-fragmentation of intermetallic crystals is a result of both acoustic streaming and acoustic cavitation-induced shock waves. The fragmentation is primarily governed by the impact of propagating shock waves produced from subharmonic cloud collapses, while the acoustic streaming is responsible for bringing the crystals into the effective cavitation region. Crystal fragmentation mostly occurs near the source as a result of shock-induced bending/torsion, where the majority of high amplitude shock waves are generated. Interestingly, the eventual breakage of floating crystals occurs by the same fatigue failure from repetitive shock wave impacts as in the case of fixed crystals [22]. However, the fragmentation, refinement and dispersion of such particulates can also be realised through the mechanism of cavitation-induced microjetting and de-agglomeration [61] and remains a subject for further research. The high-speed images (Fig. 4a, Fig. 5a and b) and the corresponding Supplementary Videos that show the fragmentation of intermetallics in real-time did not indicate any crystal breakage occurring within the cavitation zone that transpires through the mechanism other than propagating shock waves. Any such fragmentation events realised away from the horn tip maybe further related to mechanical and hydrodynamical effects induced by the cavitation bubbles, but were not observed in our study.

### 3.3 Effect of ultrasound on microstructural damage of free-floating crystals

Having revealed the main fragmentation mechanism that leads to fragmentation for free-floating crystals, we now turn our attention the micro-fragmentation response of free-floating crystals from the microstructural perspective.

**Fig. 6:**
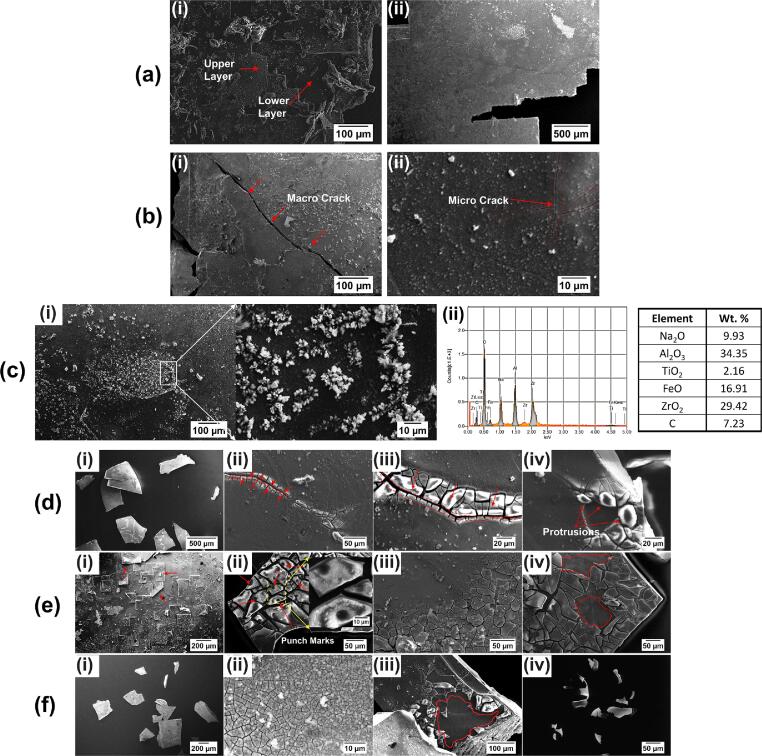
(a) SEM images of an extracted Al_3_Zr crystal showing layered and faceted morphology, (b) presence of pre-existing micro-cracks, and (c) oxides and the EDS spectrum with elemental composition. (d) SEM morphological images of cavitation damage induced after 3 sec, (e) 6 sec, and (f) 9 sec sonication, obtained at ultrasonic amplitude of 210 μm peak-to-peak.

Figure 6(a-c) displays the morphology of an extracted Al_3_Zr intermetallic crystal prior to ultrasound exposure. The untreated intermetallic exhibited a layered and faceted structure as shown in Fig. 6a (i) and 6a (ii) reflecting its growth morphology as discussed elsewhere [62]. In addition, these crystals with thicknesses in the range of 60–70 μm contained pre-existing macro- (Fig. 6b (i)) and micro-cracks (Fig. 6b (ii)) due to released residual stresses during solidification and extraction process as described in section 2.1. A considerable number of foreign particles were also found in various locations on the surface layer of the crystals, as shown in Fig. 6c (i). EDS examination of these particles indicated the presence of various oxides with sizes of 5–10 μm such as Al_2_O_3_, ZrO_2_, Na_2_O, TiO_2_, FeO (Fig. 6c (ii)) some of which might have formed during storage and material preparation while other represent indigenous oxide particles that can potentially act as an additional source of nucleation sites for the growth of Al grains [63] and primary intermetallics [64]. The surface morphology and internal defects of these crystals can have a substantial impact on their fragmentation response characteristics as previously discussed in Section 3.1. Campbell [65] has shown that primary intermetallics that form during solidification of Al melts may (actually he claims – always) nucleate onto oxide bi-films that act as internal cracks within the intermetallic particles. Therefore, the fragmentation dynamics of these extracted crystals can provide new insights into their refinement mechanism in a real melt processing environment.

A detailed post-exposure SEM examination of the single crystal after ultrasonic treatment was performed to analyse the morphology of cavitation-induced damage as shown in Fig. 6(d-f). Fig. 6d shows the morphology of intermetallic fragments after 3 sec of ultrasonic treatment. As it can be seen from Fig. 6d (i), the crystal broke into several small fragments with sizes as small as 50 μm. In addition, zooming onto the bigger fragments showed a well-defined fracture network developed along the existing micro-cracks as shown in Fig. 6d (ii) and 6d (iii). It is interesting to note that the surface of the fragment showed round protrusions 5–20 μm in size with well-defined edges, bulging out from the cracked upper layer matching their peripheral boundaries (Fig. 6d (iv)). Fig. 6e displays the morphological images of fragmented crystals after 6 sec of exposure to ultrasound. Increased ultrasound exposure led to delamination of the top layer (indicated with red arrow) as shown in Fig. 6e (i). Also, the formation of a micron-sized hierarchical crack pattern, in the range of 20–100 μm, can be observed on the upper layer of the fragmented crystal (arrowed red, Fig. 6e (ii) and 6e (iii)). The presence of plastic pits (round punch marks) of size 6 ± 2 μm can also be seen on the fractured surface as shown in Fig. 6e (ii). In addition, peeling of the cracked upper layer (as indicated with red contour) can also be seen at certain regions of the fragmented crystal as observed in Fig. 6e (iv). Fig. 6f exhibits the microstructural images of cavitation-induced damage of the primary crystal after UST for 9 sec. Further increase in the treatment duration generated more fragments of smaller size (Fig. 6f (i)) and also resulted in the formation of a finer hierarchical crack network in the range of 1–5 μm (Fig. 6f (ii)). Extensive erosion and ‘chipping off’ of the upper crystal layer can also be seen for the fragmented intermetallics as shown in Fig. 6f (iii). Individual fragments with sizes in the range of 10–50 μm, broken from the fractured surface can be seen in Fig. 6f (iv).

Strong impact pressures from collapsing bubbles generating powerful shock waves and high-speed liquid-microjets are deemed likely to be responsible for the induced fracture, surface delamination and erosion as discussed in section 3.2 and elsewhere [66], [67], [68]. In addition to emitted shock waves, these collapses also generate liquid jets with velocities in the order of 100 m/s near the crystal surface, producing water-hammer pressures *P_h_* on impact, given by, *P_h_ = ρcv* [69], where *ρc* is the density times the speed of sound in water and *v* is the liquid jet velocity, of up to 0.1 GPa sufficient to generate plastic pits (Fig. 6e (ii)) arising from multiple micro-jet impacts as previously shown in [66], [68]. Outside the cavitation zone, the crystals are less likely to be damaged by liquid jets and are mostly accompanied by stably pulsating ultrasonic bubbles that undergo distortion of interface resulting in splitting, shape and chaotic oscillations as shown in Fig. 4a (viii-ix). It has been reported that these chaotically oscillating bubbles also emit liquid jets upon splitting, but the values are substantially lower, i.e. about 1 m/s and a corresponding dynamic pressure of only 10 kPa, which is unlikely to induce any significant damage of the solid surface nearby [70]. Kim et al. [70] were first to observe the damage of a silicon wafer caused by the ultrasonically splitting bubbles. It was found that the damage was possible only if the bubble split occurred directly over the surface. Ishida et al. [71] reported that the impulsive pressure due to the emitted shock wave generated by a splitting of a spark-induced bubble when confined within the narrow surface is enough to induce microstructural damage/erosion patterns on the solid structures. Therefore, it is possible that the microstructural damage in the form of hierarchical crack patterns on the surface of fragments as shown in Fig. 6(d-f) was predominantly generated by shock waves and complemented by high-speed liquid jets produced from the periodic collapses of the cavitating cloud near the sonotrode-tip [66], [68], [72]. Whereas, chaotically oscillating ultrasonic bubbles present outside the cavitation zone may only scrub the surface of attached fragments, facilitating the removal of some exogenous non-metallic inclusions, as observed in Fig. 6c rather than damaging the surface [70], [71].

As the Al_3_Zr primary crystals in Al melt grew in steps, it is likely that repetitive exposure to cavitation bubbles induce delamination of several atomic layers, prior to cracking. The exposed layer thus became loose and then cracked owing to the stress generated by bubbles and potential discontinuities with the properties of the bulk material underneath and partially delaminated top layer. Subsequently, the loosely bonded sections of the outer layers of the crystal began to peel off, and the large cracks grew and opened up causing further breakage as it recirculated back into the cavitation zone, by induced acoustic streaming. Safonov et al. [73] previously observed that the material degradation of a multilayer brittle material by cavitation occurs in stages. It was reported that in the regions of low cavitation load, the outer layer degradation occurred through multiple impacts of cavitation bubbles inducing brittle fracture. Therefore, delamination followed by further crack formation and growth (as seen in Fig. 5b) explains the observed ‘peeling off’ of layers governing the micro-fragmentation behaviour of Al_3_Zr primary crystals, similar to the mechanism discussed elsewhere for thermal-sprayed coatings [74].

### 3.4 Crystal size reduction potential

The final step in this study was to identify the statistical distribution of the fragments based on the sonication time, which is important to control the treatment duration within the cavitation zone and optimise flow management techniques for continuous casting as recently discussed in [3], [42].

**Fig. 7:**
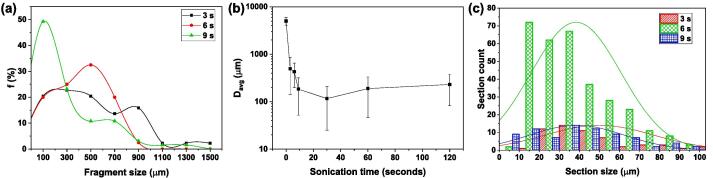
(a) Relative fragment number density vs. size distribution measured after 3, 6 and 9 sec of sonication, (b) Average crystal diameter as a function of sonication duration up to 120 sec. (c) Section size distribution and number measured after 3, 6 and 9 sec of sonication, obtained from micron-size hierarchical crack pattern images.

Figure 7a shows the size distribution of fragmented particles after sonicating for 3, 6 and 9 s based on the results of SEM imaging. As expected, the average size decreased with treatment time. With just 3 sec of treatment, almost all fragments had a size smaller than 1000 μm down from the initial size of 5 mm. The smallest crystal size from the analysis of at least 3 sets of collected fragment batches was 12 ± 1 μm with approximately 20% of fragments having sizes below 100 μm. The fragmentation potential of UST increased by almost 150% after 6 sec of sonication, however, the total number density of all the produced fragments remains approximately the same after 6 sec. With a further increase in the sonication time to 9 sec, the fragment number density increases by almost 200% with more than 50% of the fragments being smaller than 100 μm.

Figure 7b shows the fragmentation potential of UST for intermetallic crystals represented in terms of average fragment size with respect to ultrasonic treatment durations up to 120 sec. A significant decrease in the fragment size was visible after 3 sec of treatment, indicating the severity of particle breakage. The mean size reduced by more than 10 times from the initial crystal dimension showing high propensity of intermetallics to fragment under the combined action of acoustic cavitation and acoustic streaming. The average size dropped to almost 500 μm within the first 6 sec of treatment. The slope of the fragment size with respect to sonication time, however, only slightly decreased thereafter reaching a plateau after 9 sec indicating a stabilised fragmentation process. This was possibly because the fragments were not sufficiently large to achieve the required flexing/bending upon shock wave incidence, as described previously. The average size of fragments decreased to 100 μm and remained constant until 120 sec of sonication duration. The majority of the fragments of sizes<100 μm produced after 9 sec of treatment, were more likely to be the result of delamination and breakage of the fractured crystal surface, as observed in Fig. 6f. The results indicate that the effective treatment time of intermetallics within the active fragmentation zone (defined in section 3.1) in water medium should be in the range of 10–15 sec which coincides well with the time required for efficient intermetallic fragmentation upon casting of Al alloys [3], [33]. It is important to note here that in a real casting processes, the choice of effective treatment time will also depend on the process scale dimensions and various other important factors such as casting speed, horn dimension etc.

As identified in Fig. 6(d-f), many of the large broken fragments also showed the presence of micron-size crack patterns in the form of grains/platelets and, with the increase in sonication time, a much finer crack network was generated on the exposed surface of the fragments with the individual sections measuring 100 μm or less. It is expected that these tiny sections will eventually break off from the surface and play the role of effective nuclei within the Al melt, further promoting heterogeneous nucleation of new Al grains. With this in mind, we also analysed these crack patterns generated after 3, 6 and 9 sec of treatment.

Figure 7c shows the section size and its number distribution obtained from micron-size hierarchical crack network pattern for different sonication periods as discussed in relation to Fig. 6(d-f). It can be seen that overall section count increased with the treatment time from 3 sec to 6 sec by a factor of 7 and then subsequently decreased for the 9 sec sonication for sections with sizes between 10 and 100 μm. The number of the individual sections with the size below 10 μm however, increased by a factor of almost 8, from 3 sec to 9 sec. The average size of the grains formed after 3 sec decreases from approximately 50 μm to 38 μm after 6 sec and then remains almost unchanged even with 9 sec with fewer number of grains ranging from 10 to 100 μm. It might be that with a further increase of treatment time beyond 6 sec, the resulting cracked sections broke out from the damaged layer of the intermetallic crystal, thereby increasing the number of smaller size fragments as shown in Fig. 7a.

A similar qualitative trend in particle size reduction and number distribution as a result of sono-fragmentation has been reported in a number of studies [7], [12], [55], [75], [76]. Although the effect of sono-fragmentation on the particle size reduction was observed at different operating frequencies, most of the papers reported a significant size reduction for low sonication frequencies (circa 20 kHz), where the effect of shock wave was more pronounced, making it ideal for treating liquid metals as compared to higher frequencies [8], [76], [77], [78]. In the case of UST of real Al alloys, however, such big crystals 5–7 mm in dimensions would be not formed under normal casting conditions. Therefore, although fragmentation of intermetallics in Al melts under cavitation does happen and has been observed experimentally (ex-situ) before, the specific results obtained from the fragment size distribution are confined to the experimental conditions used, i.e. crystal/particulate breakage in water.

**Fig. 8:**
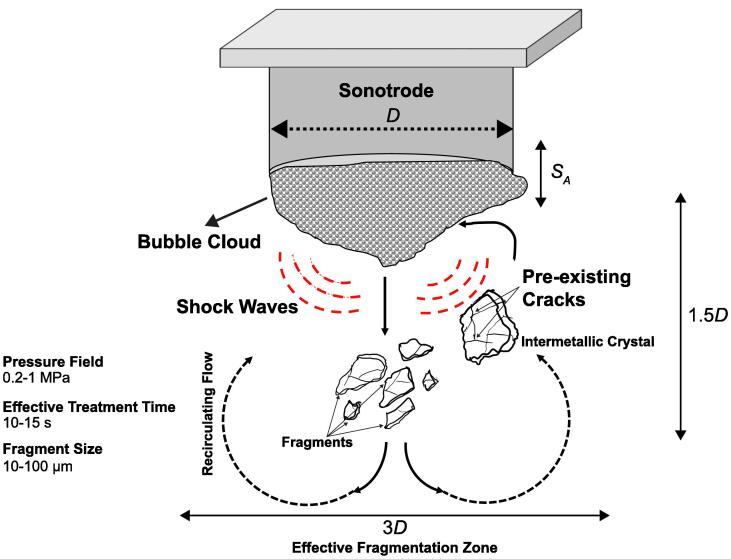
Schematic representation of free-floating crystal fragmentation mechanism showing the extent of effective treatment domain.

Figure 8 shows a schematic of the free-floating intermetallic fragmentation mechanism summarizing the fragmentation observations made in our study. The continuous interaction of the floating intermetallics with the periodic shock wave emissions from high-energy collapses of the cavitation cloud with the pressure field in the range of 0.2–1 MPa facilitated by the recirculating acoustic flow results in the formation of numerous small fragments (10–100 μm) within just a few seconds of treatment (10–15 sec). The effective fragmentation zone can extend up to 1.5 times the diameter of the ultrasonic source in longitudinal (Y-axis) and up to 3 times in transverse (X-axis) direction, as observed in Fig. 2b. The rapid reduction in fragment size can be due to the brittle nature of the intermetallics and the presence of various micro-cracks and defects making them more susceptible to fragmentation. With the progression in fragmentation, the possibility of fragments to further break up is expected to reduce, making the subsequent rate of fragmentation slower as is demonstrated upon UST longer than 9 sec. This may be a result of the smaller aspect ratio of remaining fragments increasing their inherent strength.

In this work, temporal and spatial statistics for crystal fragmentation were generated in water for fixed and free-floating conditions important for identifying the optimum treatment duration and domain for a larger scale real melt processing units. It is likely that the crystal fragmentation will be even more pronounced in liquid aluminium leading to increased particle number and even smaller particles compared to observations made here, in water. Tzanakis et al. [21], [79] observed that the cavitation dynamic response in liquid Al is almost twice as intense in terms of acoustic pressure field generated. Moreover, the higher density of liquid Al will prevent fast sedimentation of the primary crystals increasing their exposure to further fragmentation. In real ultrasonic melt processing, the estimation of treatment time or residence time becomes very important in order to ensure that the whole liquid volume passing through the cavitation zone is uniformly treated [80]. Therefore, based on the temporal and spatial characteristics of the crystal fragmentation as described in this paper, the optimum size of the treatment vessel, size of sonotrode and sonication time can be estimated and used for numerical modelling of ultrasonic processing.

## 4 Conclusions

In this study, the fragmentation potential of fixed and free-floating primary intermetallics was examined based on the effective spatial and temporal domain treatment characteristics that maximize the extent of crystal refinement when subjected to ultrasonic treatment. Following investigations of fragmentation of fixed and free-floating intermetallics through in-situ high-speed imaging, acoustic measurements, and post-exposure SEM study of the crystal fragments, the following conclusions can be drawn:

1. Effective cavitation zone mapping experiments for fixed intermetallics reveal that time required for first breakage of a crystal mostly lies in the range of 20–100 ms irrespective of its position as long as crystal lies within the actual treatment zone (5 ≤ $\left|x\right|$ ≤ 30; 5 ≤ $\left|y\right|$ ≤ 30 mm) and the developed pressure field that spans from 200 to 1300 kPa for all the input powers (20%, 60%, and 100%). However, what regulates the breakage of the studied crystals is the bubble volume fraction that surpasses the developed pressure field and cushions the propagating shock waves (predominantly responsible for the fragmentation). Therefore, the crystal fragmentation time may be delayed even by 2–3 times, as seen in the case of 100% power.
2. For the first time high-speed filming captured the dynamic interaction of shock waves with floating intermetallic particles that led to their fragmentation. Repeated fragmentations are induced as the crystals recirculate with the acoustic streaming generated from the vibrating sonotrode.
3. Microscopic observations of fragmented free-floating crystals show the formation of protrusions, plastic pits and micron-sized hierarchical crack network on the surface of the fragments. The size of crack network decreases with the increasing treatment time, beyond 6 sec and are in the range of 5 to 50 μm. SEM imaging of the fragmented crystals suggests that the intermetallic fracture occurs through delamination/fracture of the exposed top layer. The fragmentation is likely to occur within the cavitation zone (close to bubble cloud) upon repetitive shock wave impacts. The smaller fragments, with sizes below 100 μm, are most likely produced through the breakage of the delaminated surface layer.
4. The size reduction potential of intermetallics assessed by the post-treatment fragment analysis (using SEM) indicates that the average fragment size and relative density decreases and increases, respectively, with the extended sonication period. The crystal fragmentation initially occurs rapidly, and subsequently slows down, generating crystals smaller than 100 μm in just 9 sec of treatment.

Future work should focus on understanding of crystal fragmentation dynamics in a real melt environment through in-situ X-ray synchrotron imaging and measuring the mechanical properties of Al_3_Zr crystals near the liquidus temperature of the alloy through advanced high temperature nanoindentation. These experiments can be further coupled with the current results and used for numerical modelling of crystal fragmentation in various Al alloys and optimisation of the UST process conditions.

### CRediT authorship contribution statement

**Abhinav Priyadarshi:** Conceptualization, Methodology, Data curation, Software, Validation, Formal analysis, Investigation, Resources, Writing – original draft, Writing – review & editing. **Mohammad Khavari:** Software, Formal analysis, Investigation. **Shazamin Bin Shahrani:** Formal analysis, Investigation. **Tungky Subroto:** Resources. **Lukman A. Yusuf:** Formal analysis, Investigation. **Marcello Conte:** Resources. **Paul Prentice:** Supervision, Writing – review & editing. **Koulis Pericleous:** Writing – review & editing, Supervision, Funding acquisition. **Dmitry Eskin:** Writing – review & editing, Resources, Supervision, Funding acquisition. **Iakovos Tzanakis:** Conceptualization, Methodology, Writing – review & editing, Supervision, Funding acquisition.

## Declaration of Competing Interest

The authors declare that they have no known competing financial interests or personal relationships that could have appeared to influence the work reported in this paper.
