# Supplementary material for: In-situ observations and acoustic measurements upon fragmentation of free-floating intermetallics under ultrasonic cavitation in water
Source: Ultrason Sonochem. 2021 Nov 2;80:105820. doi: 10.1016/j.ultsonch.2021.105820 (PMC8591476; doi:10.1016/j.ultsonch.2021.105820)
Supplement: Supplementary FIgures [file mmc1.docx]

**In-situ observations and acoustic measurements upon fragmentation of free-floating intermetallics under ultrasonic cavitation in water**

Abhinav Priyadarshi ^a,*^, Mohammad Khavari ^a^_,_ Shazamin Bin Shahrani ^a^, Tungky Subroto ^b^, Lukman A. Yusuf ^c^, Marcello Conte ^d^, Paul Prentice ^c^, Koulis Pericleous ^e^, Dmitry Eskin ^b,f^, Iakovos Tzanakis ^a,g^

^a^ Faculty of Technology, Design and Environment, Oxford Brookes University, Oxford OX33 1HX, United Kingdom

^b^ Brunel Centre for Advance Solidification Technology (BCAST), Brunel University London, Uxbridge UB8 3PH, United Kingdom

^c^ Cavitation Laboratory, School of Engineering, University of Glasgow, Glasgow G12 8QQ, United Kingdom

^d^ Anton Paar TriTec SA, Vernets 6, 2035 Corcelles, Switzerland

^e^ Computational Science and Engineering Group (CSEG), Department of Mathematics, University of Greenwich, London SE10 9LS, United Kingdom

^f^ Tomsk State University, Tomsk 634050, Russia

^g^ Department of Materials, University of Oxford, Oxford OX1 3PH, United Kingdom

**Supplementary Figures**

**
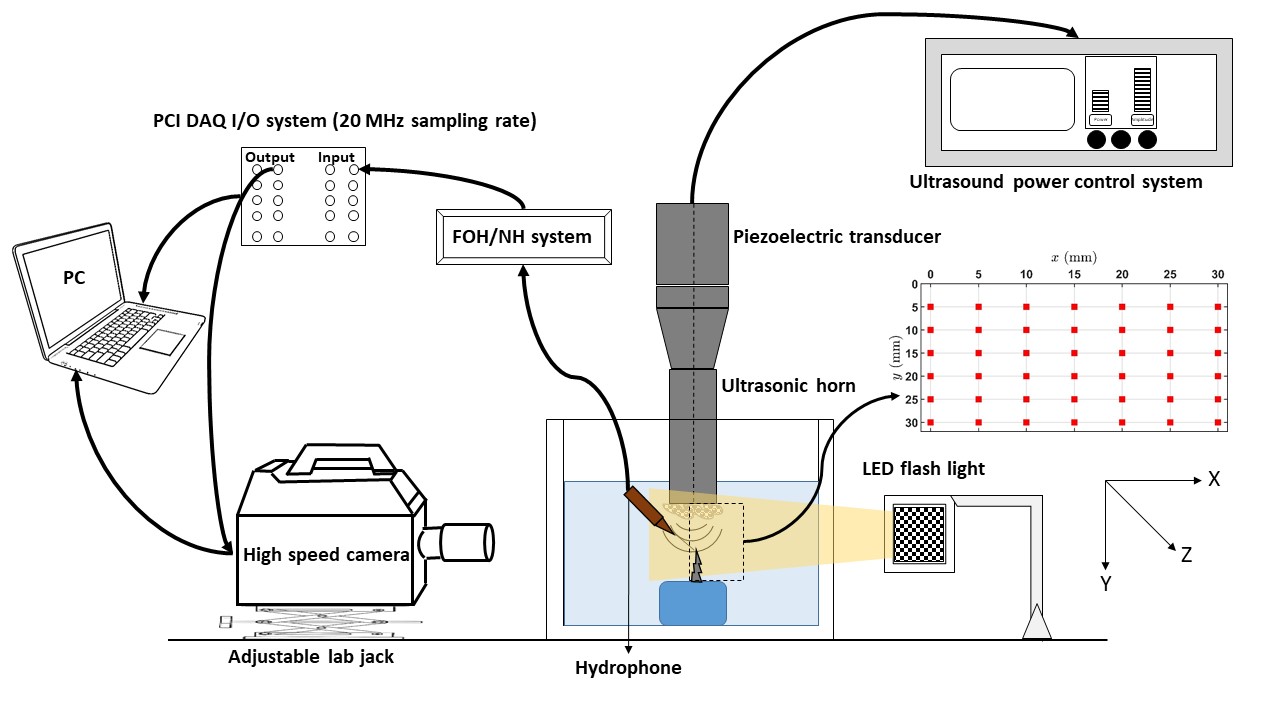
**

**Fig. S1.** Experimental setup to identify the effective treatment zone via crystal fragmentation time and acoustic pressure mapping in transverse (X-axis) and longitudinal (Y-axis) direction.


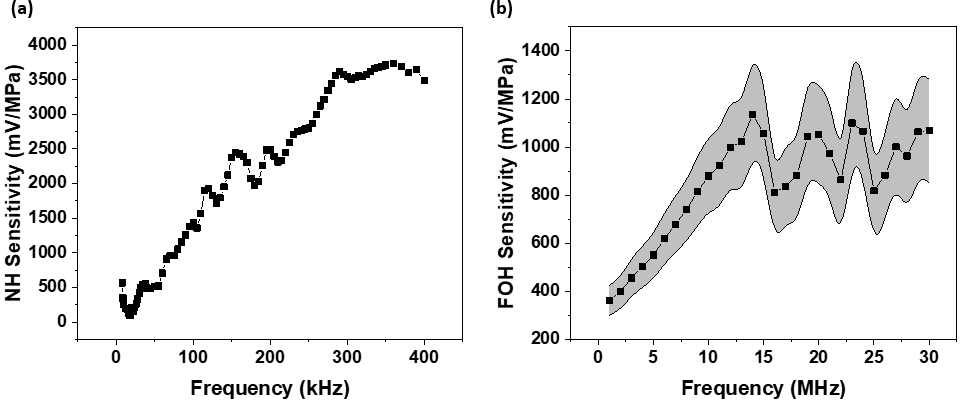


**Fig. S2.** Sensitivity of (a) NH calibrated between 8-400 kHz, (b) FOH calibrated between 1-30 MHz bandwidth frequencies.


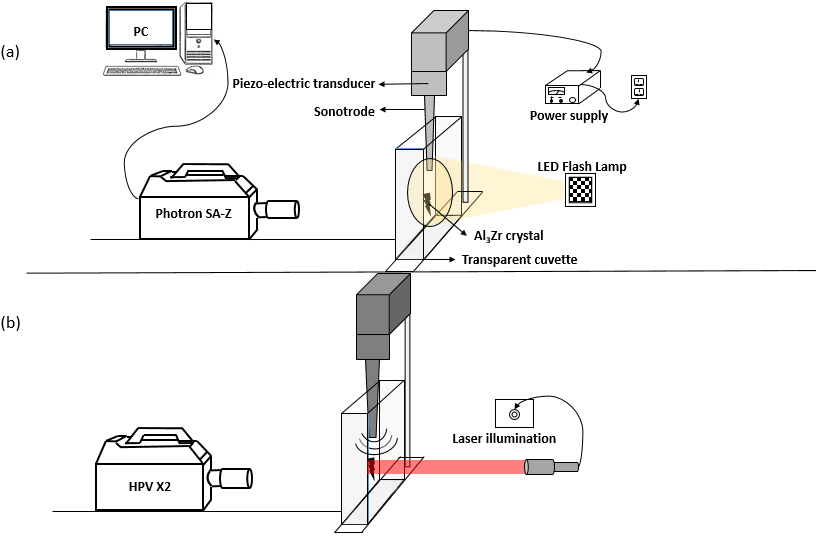


**Fig. S3.** Schematic of a high-speed imaging experimental setup to (a) capture the sequence of fragmentation process, spatial and temporal distribution as well as the dynamic size reduction of the fragments under light illumination; (b) capture sequence of crystal fragmentation by shock waves under laser illumination.
